# Supplementary material for: Estimating survival parameters under conditionally independent left truncation
Source: Pharm Stat. 2022 Mar 9;21(5):895–906. doi: 10.1002/pst.2202 (PMC9545094; doi:10.1002/pst.2202)
Supplement: Supplementary file 1 — APPENDIX S1: Supporting information. [file PST-21-895-s001.pdf]

# Estimating survival parameters under conditionally independent left truncation

Arjun Sondhi
Flatiron Health

## Supplement

In this appendix, we sketch a proof of consistency for the weighted and risk set adjusted Kaplan-Meier estimator, given appropriate density ratio weights. Recall that we define $T$ as the survival time, $C$ as the censoring time, and $E$ as the entry time. We observe $Y = \min(T, C)$ conditional on $Y > E$, with $\delta = I(T \leq C)$ as the event indicator. The observed event times are $x_j, j = 1, ..., m$. $Z$ is a vector of confounders such that $Y \perp E|Z$

We begin by deriving the product form of the survival probability, given independently right-censored data.

$$
\begin{aligned}
P(T \geq x_k) &= \Pi_{j=0}^{k-1} S(x_{j+1}) \\
&= \Pi_{j=0}^{k-1} P(T \geq x_{j+1} | T \geq x_j) \\
&= \Pi_{j=0}^{k-1} [1 - P(T < x_{j+1} | T \geq x_j)] \\
&= \Pi_{j=0}^{k-1} [1 - P(T \in [x_j, x_{j+1}) | T \geq x_j)] \\
&= \Pi_{j=0}^{k-1} [1 - P(T = x_j | T \geq x_j)] \\
&= \Pi_{j=0}^{k-1} [1 - P(Y = x_j, \delta = 1 | Y \geq x_j)] \\
&= \Pi_{j=0}^{k-1} [1 - F(x_j)]
\end{aligned}
$$

Then, the weighted and risk set adjusted Kaplan-Meier estimator for each term in this expression is given by

$$
\hat{F}(x_j) = \frac{\sum_{i=1}^{n} I(E_i \leq x_j, Y_i = x_j)\delta_i w_i}{\sum_{i=1}^{n} I(E_i \leq x_j \leq Y_i) w_i},
$$

where $w_i$ is a weight for subject $i$. Specifically,

$$
w_i = \frac{\pi(z_i)}{\pi(z_i | y_i > e_i)},
$$

the density ratio comparing the covariate distributions of the non-truncated and left truncated datasets.

To show consistency, we compute the expectations of the numerator and the denominator. First the

expectation of the numerator is:

$$
\begin{aligned}
&\mathbb{E}\left[\sum_{i=1}^{n} I(E_i \leq x_j, Y_i = x_j)\delta_i w_i | Y_i > E_i\right] \\
&= \sum_{i=1}^{n} \mathbb{E}[I(E_i \leq x_j, Y_i = x_j)\delta_i w_i | Y_i > E_i] \\
&= \sum_{i=1}^{n} \int I(E_i \leq x_j, Y_i = x_j)\ \delta_i\ w_i\ \pi(y_i, e_i, z_i | y_i > e_i)\ d(y, e, z) \\
&= \sum_{i=1}^{n} \int I(E_i \leq x_j, Y_i = x_j)\ \delta_i\ w_i\ \pi(y_i, e_i | z_i, y_i > e_i)\ \pi(z_i | y_i > e_i)\ d(y, e, z) \\
&= \sum_{i=1}^{n} \int I(E_i \leq x_j, Y_i = x_j)\ \delta_i\ w_i\ \pi(y_i | z_i, y_i > e_i)\ \pi(e_i | z_i, y_i > e_i)\ \pi(z_i | y_i > e_i)\ d(y, e, z) \\
&= \sum_{i=1}^{n} \int I(E_i \leq x_j, Y_i = x_j)\ \delta_i\ w_i\ \pi(z_i | y_i > e_i)\ \pi(y_i | z_i)\ \pi(e_i | z_i)\ d(y, e, z) \\
&= \sum_{i=1}^{n} \int I(E_i \leq x_j, Y_i = x_j)\ \delta_i\ \pi(z_i)\ \pi(y_i | z_i)\ \pi(e_i | z_i)\ d(y, e, z) \\
&= \sum_{i=1}^{n} \int I(E_i \leq x_j, Y_i = x_j)\ \delta_i\ \pi(y_i, e_i, z_i)\ d(y, e, z) \\
&= \sum_{i=1}^{n} \mathbb{E}[I(E_i \leq x_j, Y_i = x_j)\delta_i] \\
&= \sum_{i=1}^{n} \mathbb{E}\left(\mathbb{E}[I(E_i \leq x_j, Y_i = x_j)\delta_i | Z]\right) \\
&= \sum_{i=1}^{n} \mathbb{E}\left(\mathbb{E}[I(E_i \leq x_j) I(Y_i = x_j)\delta_i | Z]\right) \\
&= \sum_{i=1}^{n} \mathbb{E}[I(E_i \leq x_j) I(Y_i = x_j)\delta_i] \\
&= \sum_{i=1}^{n} P(E_i \leq x_j) P(Y_i = x_j, \delta_i = 1) \\
&= nP(E \leq x_j) P(Y = x_j, \delta = 1)
\end{aligned}
$$

Similarly, for the denominator, we can obtain:

$$
\begin{aligned}
&\mathbb{E}\left[\sum_{i=1}^{n} I(E_i \leq x_j \leq Y_i)\ w_i | Y_i > E_i\right] \\
&= \sum_{i=1}^{n} \mathbb{E}[I(E_i \leq x_j \leq Y_i)] \\
&= \sum_{i=1}^{n} \mathbb{E}(\mathbb{E}[I(E_i \leq x_j \leq Y_i) | Z]) \\
&= \sum_{i=1}^{n} \mathbb{E}(\mathbb{E}[I(E_i \leq x_j) I(Y_i \geq x_j) | Z]) \\
&= \sum_{i=1}^{n} \mathbb{E}[I(E_i \leq x_j) I(Y_i \geq x_j)] \\
&= nP(E_i \leq x_j) P(Y_i \geq x_j)
\end{aligned}
$$

Therefore, by applying the continuous mapping theorem:

$$\hat{F}(x_j) \longrightarrow \frac{nP(E \leq x_j)P(Y = x_j, \delta = 1)}{nP(E \leq x_j)P(Y \geq x_j)} = P(Y = x_j, \delta = 1 | Y \geq x_j),$$

as required.
